# Mental health improvement after the COVID-19 pandemic in individuals with psychological distress

**DOI:** 10.1038/s41598-024-55839-3

**Published:** 2024-03-07

**Authors:** Mario Reutter, Katharina Hutterer, Marthe Gründahl, Dominik Gall, Udo Dannlowski, Katharina Domschke, Elisabeth J. Leehr, Tina B. Lonsdorf, Ulrike Lueken, Andreas Reif, Miriam A. Schiele, Peter Zwanzger, Paul Pauli, Grit Hein, Matthias Gamer

**Affiliations:** 1 Department of Psychology I, Julius-Maximilians-University Würzburg, Marcusstr. 9-11, 97070 Würzburg, Germany; 2 Department of Psychiatry, Psychosomatics and Psychotherapy, Clinical Anxiety Research, Center of Mental Health, University of Würzburg, Würzburg, Germany; 3 Translational Social Neuroscience Unit, Department of Psychiatry, Psychosomatics and Psychotherapy, Center of Mental Health, University of Würzburg, Würzburg, Germany; 4 Institute for Translational Psychiatry, University of Münster, Münster, Germany; 5 Department of Psychiatry and Psychotherapy, Faculty of Medicine, Medical Center – University of Freiburg, University of Freiburg, Freiburg, Germany; 6 Institute for Systems Neuroscience, University Medical Center Hamburg-Eppendorf, Hamburg, Germany; 7 Department of Psychology, Biological Psychology and Cognitive Neuroscience, University of Bielefeld, Bielefeld, Germany; 8 Department of Psychology, Humboldt-Universität zu Berlin, Berlin, Germany; 9 Department of Psychiatry, Psychosomatic Medicine and Psychotherapy, University Hospital, Goethe University Frankfurt, Frankfurt, Germany; 10 Fraunhofer Institute for Translational Medicine and Pharmacology ITMP, Frankfurt, Germany; 11 kbo-Inn-Salzach-Klinikum, Clinical Center for Psychiatry, Psychotherapy, Psychosomatic Medicine, Geriatrics and Neurology, Wasserburg/Inn, Germany; 12 Department of Psychiatry, Ludwig-Maximilian-University Munich, Munich, Germany; 13 German Center for Mental Health (DZPG), Partner Site Berlin/Potsdam, Berlin, Germany

**Keywords:** Psychology, Public health

## Abstract

The COVID-19 pandemic and associated countermeasures had an immensely disruptive impact on people’s lives. Due to the lack of systematic pre-pandemic data, however, it is still unclear how individuals’ psychological health has been affected across this incisive event. In this study, we analyze longitudinal data from two healthy samples (*N* = 307) to provide quasi-longitudinal insight into the full trajectory of psychological burden before (baseline), during the first peak, and at a relative downturn of the COVID-19 pandemic. Our data indicated a medium rise in psychological strain from baseline to the first peak of the pandemic (*d* = 0.40). Surprisingly, this was overcompensated by a large decrease of perceived burden until downturn (*d* =  − 0.93), resulting in a *positive* overall effect of the COVID-19 pandemic on mental health (*d* = 0.44). Accounting for this paradoxical positive effect, our results reveal that the post-pandemic increase in mental health is driven by individuals that were already facing psychological challenges before the pandemic. These findings suggest that coping with acute challenges such as the COVID-19 pandemic can stabilize previously impaired mental health through reframing processes.

## Introduction

The world has been significantly changed by the COVID-19 pandemic. Until Nov 17th 2023, there have been over 771 million confirmed infections with COVID-19 and almost 7 million associated deaths^1^. The nations of the world reacted in different ways to this public threat, with many governments issuing recommendations for physical distancing and even legally enforcing lockdowns^2^. In Germany, for example, the trajectory of the pandemic is frequently divided into a total of four waves of quickly rising cases in spring 2020, winter 2020/2021, spring 2021, and winter 2021/22^3,4^. The German government responded to the first two waves with nationwide lockdowns^5,6^, which were replaced by local measures during the third wave depending on the number of infections per time within a region^7^. Prior to and during the fourth wave, citizens were obliged to provide a certificate of vaccination against, recovery from, or a negative test of COVID-19 in order to participate in public activities and even working life^8^.

In addition to physical danger from infections, the COVID-19 pandemic constitutes a threat for mental health due to ongoing stress and uncertainty. Researchers attribute an increase of more than 25% in depressive and anxiety symptoms to the pandemic, with local infection rates and restrictions in personal mobility exhibiting the largest predictive power^9^. This rise in psychological distress also affected healthy individuals^10^, albeit to a lesser degree (^11^; but see^12^). Risk was found to be higher in females and young individuals^9,13,14^, which was reflected in these groups exhibiting most frequent help seeking behavior^15^. Also at jeopardy were people with financial insecurity^13,14,16^ and inadequate physical space during periods of lockdown isolation^17^. Moreover, individuals with a COVID-19 diagnosis within their social environment during the first wave^18^ or those who perceived the danger of COVID-19 to be higher^19^ reported elevated anxiety during the pandemic. On the other hand, social contacts (especially offline but also online) were identified as a buffer against deprivations of mental health^16,17^ because they reduce loneliness^20,21^. Also, certain stress appraisals and coping strategies have been identified as protectors of mental well-being during the pandemic^22^.

Adverse effects of the COVID-19 pandemic on mental health were particularly pronounced in individuals who already suffered from mental impairments before the outbreak of the pandemic^11,13,15^. For example, a lack of exposure to social situations may have contributed to the maintenance of symptomatology within individuals suffering from social anxiety^19,23,24^. Previous experiences of childhood trauma and other threatening events can also increase an individual’s vulnerability for the negative effects of subsequent adverse events^25,26^ such as the COVID-19 pandemic^27,28^. Note that the individual response to adverse life events can be positively affected by coping and emotion-regulatory strategies^26^, including self-efficacy^29,30^ and the use of adaptive (e.g., cognitive reappraisal) rather than maladaptive (e.g., suppression) cognitive emotion regulation strategies^15,31–33^. In summary, the COVID-19 pandemic and its countermeasures exuded a complex pattern of effects on physical and mental health, and factors shaping human stress resilience during the pandemic in the short and long run constitute a central research focus^34,35^.

One aspect that complicates research on the psychological burden of the COVID-19 pandemic is its sudden onset. Consequently, there are only few longitudinal studies with pre-pandemic baselines (for an overview, see^36^; for more recent studies with pre-pandemic baselines and longer follow-up periods, see^16,22,37^). Thus, it is difficult to assess the influence of the pandemic on people’s mental health since effects from before and during this period are conflated. Even studies with baselines in early 2020, i.e., prior to local hotspots and lockdowns in most countries, face the problem that the virus was already on the news, instilling worry for some individuals while others may have been completely unaffected by a threat that seemed still latent at the time. This uncertainty of individual pre-pandemic burden may explain inconsistencies between different studies with respect to the psychological impact of the COVID-19 pandemic: While average effects were described as relatively small in a meta-analysis by Prati and Mancini^36^, the authors noted that there is substantial heterogeneity between different investigations with respect to mental health symptoms like anxiety and depression that could not be explained by various moderators such as local death rate, extent of lockdowns, or sample demographics.

To overcome this problem of sparse longitudinal data on the impact of the COVID-19 pandemic on mental health, we used a novel approach to combine two different samples to reconstruct a (quasi-)longitudinal trajectory of psychological burden, which was calculated from questionnaires assessing different symptoms related to anxiety, worry, and depression. Using this aggregated outcome measure, we investigate the role of pre-pandemic strain on changes in mental health from before the COVID-19 pandemic across its first peak to a relative downturn in fall 2021. This approach allows to characterize the impact of the pandemic on psychological burden and to identify protective and risk factors on individual trajectories. Relative to the pre-pandemic baseline, we expected psychological burden to increase during the first pandemic peak and to partially recover at pandemic downturn. Furthermore, we hypothesized that protective factors (self-efficacy and adaptive emotion regulation strategies) would dampen this trajectory while risk factors (social anxiety, maladaptive emotion regulation strategies, and traumatic or adverse life events) would aggravate it.

## Results

### Pre-pandemic burden

Before pandemic onset, anxiety sensitivity averaged to 13.8 (*SD* = 8.81, *range* = 0–48), worry to 41.8 (*SD* = 10.5, *range* = 16–77), and trait anxiety to 35.2 (*SD* = 8.62, *range* = 20–66). Social anxiety was comparably low (*mean* ± *SD*: *SPAI* = 35.4 ± 16.7; *LSAS* = 23.5 ± 15.3) and self-efficacy was average (*GSE* = 29.6 ± 3.63; cf.^38^). Concerning emotion regulation, we observed a mean of 18.1 (*SD* = 4.60, possible values from 8 to 40) for maladaptive strategies, 26.4 (*SD* = 5.03, possible values from 8 to 40) for adaptive strategies, and 7.21 (*SD* = 1.96, possible values from 2 to 10) for acceptance. Of our sample, 12.1% reported (at least moderate) childhood trauma^39^ with an average of 1.34 (*SD* = 1.27) threatening experiences and 9.38 (*SD* = 10.8) adverse life events. None of these values were significantly different from individuals who stopped participation during pandemic downturn (|*t*|s ≤ 1.06, *p*s ≥ 0.288, *d*s ≤ 0.07), indicating no selective attrition^40^.

### Group-level trajectory

**Figure 1:**
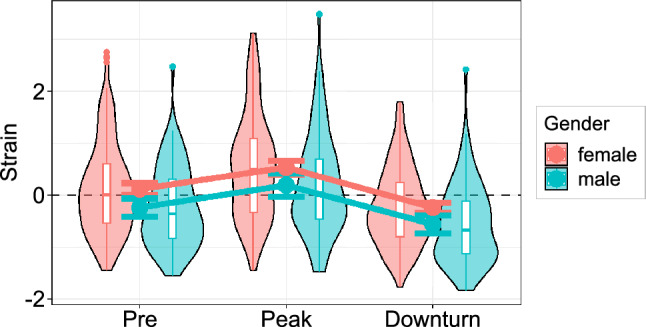
Trajectories of psychological strain as a function of time and gender. Trajectories of psychological strain are shown for females (red, *N* = 230) and males (blue, *N* = 77) before, at the peak, and during abatement of the COVID-19 pandemic. All values were *z*-standardized using the pre-pandemic mean and standard deviation. Error bars indicate 95% confidence intervals of between-subject estimates. Boxplots denote 1st, 2nd, and 3rd quartiles with whiskers extending 1.5 inter-quartile ranges or until the most extreme data point has been reached. Data points beyond the whiskers are plotted individually.

To investigate the general trajectory of psychological *strain* across the COVID-19 pandemic, we calculated a mixed effects ANOVA with *time* (pre, peak, downturn) as within-subject factor and the between-subject predictors *gender*, *age*, and *gap* (between the first and last assessment). The effect of *time* was highly significant (*F*(1.77, 529.13) = 54.54, *p* < 0.001, η_p_^2^ = 0.15) and is described by a significant rise in *strain* from pre to peak pandemic (*t*(306) = 7.07, *p* < 0.001, *d* = 0.40 [0.29; 0.52]), which was followed by an even sharper decline from peak to downturn (*t*(306) =  − 16.23, *p* < 0.001, *d* =  − 0.93 [− 1.06; − 0.79]) that resulted in values even below the pre-pandemic baseline (*t*(306) =  − 7.73, *p* < 0.001, *d* =  − 0.44 [− 0.56; − 0.32]). We also found a significant effect of *gender* (*F*(1, 299) = 5.50, *p* = 0.020, η_p_^2^ = 0.02) with higher *strain* being reported across all assessments by females (*z* = 0.14) compared to males (*z* =  − 0.20). Other effects did not reach statistical significance (*F*s ≤ 2.46, *p*s ≥ 0.117). The extent of psychological strain in females and males at the different time points is depicted in Fig. 1.

### Moderators

In subsequent analyses, we tested the influence of different pre-pandemic risk factors (social anxiety, childhood trauma, and life events) and resources (self-efficacy and coping strategies) on the trajectory of self-reported psychological strain.

#### Social anxiety

**Figure 2:**
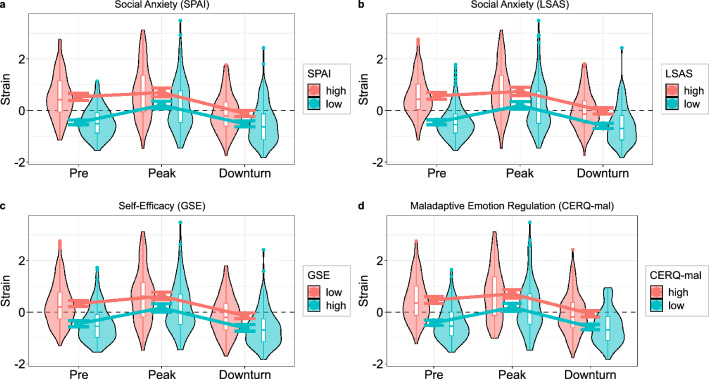
Risk factors exhibiting similar impact on the trajectory of psychological strain. Risk factors include high social anxiety (**a,b**), low self-efficacy (**c**), and high maladaptive emotion regulation strategies (**d**). All risk factors were associated with elevated baseline *strain* prior to pandemic onset but also with a less severe increase until pandemic peak. Nevertheless, people with elevated risk factors reported consistently greater strain across all time points. Risk factors were analyzed as continuous variables but are depicted as median splits for simplicity. Error bars indicate 95% confidence intervals of between-subject estimates. Boxplots denote 1st, 2nd, and 3rd quartiles with whiskers extending 1.5 inter-quartile ranges or until the most extreme data point has been reached. Data points beyond the whiskers are plotted individually.

Considering social anxiety as a risk factor, we found almost identical effects for the *SPAI* and *LSAS*, presumably due to the high correlation between questionnaires (*r* = 0.76, *p* < 0.001). Social anxiety showed a significant main effect (*SPAI*: *F*(1, 291) = 42.20, *p* < 0.001, η_p_^2^ = 0.13; *LSAS*: *F*(1, 291) = 53.44, *p* < 0.001, η_p_^2^ = 0.16), which denotes a positive correlation between social anxiety and *strain* at all time points (*SPAI*: *r*s ≥ 0.304; *LSAS*: *r*s ≥ 0.299). The interaction of social anxiety and *time* was also significant (*SPAI*: *F*(1.77, 515.44) = 12.02, *p* < 0.001, η_p_^2^ = 0.04; *LSAS*: *F*(1.78, 518.08) = 9.75, *p* < 0.001, η_p_^2^ = 0.03): The pre-pandemic *strain* was higher for participants who also reported stronger symptoms of social anxiety (*SPAI*: *r* = 0.68, *p* < 0.001; *LSAS*: *r* = 0.67, *p* < 0.001). Individuals with greater social anxiety, however, experienced a *less* pronounced rise in *strain* until the peak of the pandemic (*SPAI*: *r* =  − 0.27; *LSAS*: *r* =  − 0.26) followed by a decline to the relative downturn that was independent of social anxiety (*SPAI*: *r* = 0.00; *LSAS*: *r* = 0.03; see Fig. 2a,b). Only for the *SPAI*, we additionally observed a small but significant interaction with *gender* (*F*(1, 291) = 5.21, *p* = 0.023, η_p_^2^ = 0.02) that was driven by the correlation between *SPAI* and the average *strain* across all time points being higher for women (*r* = 0.59, *p* < 0.001) than for men (*r* = 0.38, *p* < 0.001). Other interactions did not reach statistical significance (*SPAI*: *F*s ≥ 1.43, *p*s ≤ 0.233; *LSAS*: *F*s ≥ 1.74, *p*s ≤ 0.189).

#### Self-efficacy

For self-efficacy (*GSE*), similar results as for social anxiety were observed (Fig. 2c). We found a main effect of *GSE* (*F*(1, 290) = 28.22, *p* < 0.001, η_p_^2^ = 0.09), reflecting an increase in *strain* with decreasing self-efficiency across all time points (*r*s ≤  − 0.21). Additionally, an interaction of *GSE* and *time* was found (*F*(1.78, 515.76) = 9.89, *p* < 0.001, η_p_^2^ = 0.03). Pre-pandemic *strain* was greater for individuals with less self-efficacy (*r* = 0.56, *p* < 0.001) but they also experienced a smaller increase during pandemic peak (*r* = 0.26, *p* < 0.001). The change from peak to downturn, however, was independent of self-efficacy (*r* =  − 0.06, *p* = 0.261).

#### Emotion regulation

Maladaptive emotion regulation strategies (*CERQ-mal*) showed the same pattern as the previous risk factors (Fig. 2d). There was a main effect of *CERQ-mal* (*F*(1, 291) = 38.34, *p* < 0.001, η_p_^2^ = 0.12) that was reflected by positive associations with *strain* across all time points (*r*s ≥ 0.23). We also observed an interaction with *time* (*F*(1.80, 523.46) = 11.47, *p* < 0.001, η_p_^2^ = 0.04): While baseline *strain* was elevated for participants with maladaptive emotion regulation strategies (*r* = 0.62, *p* < 0.001), the rise during the first pandemic peak was less pronounced for these individuals (*r* =  − 0.29, *p* < 0.001). The following decline until downturn was yet again independent of maladaptive emotion regulation strategies (*r* = 0.08, *p* = 0.145).

**Figure 3:**
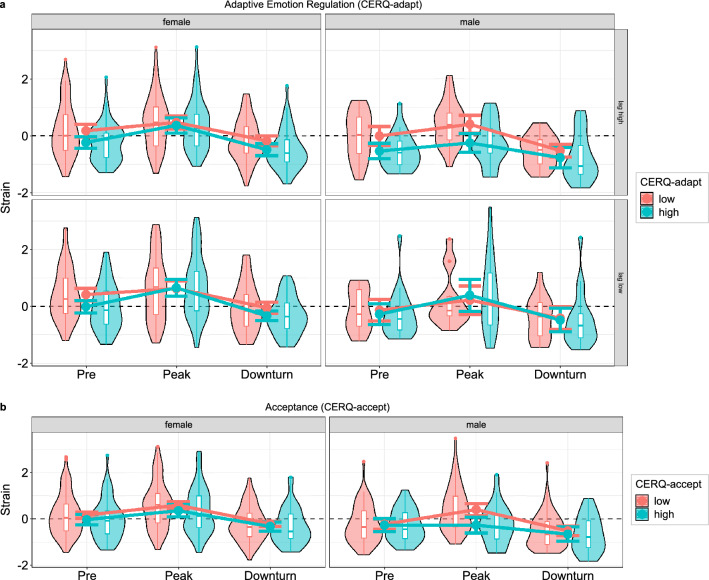
Interactive effects of emotion regulation and gender on psychological strain. For adaptive emotion regulation strategies (**a**), men with higher values experienced a smaller rise in strain until pandemic peak but only if the time gap between first and second assessment was also comparably high (blue line in top right subplot). Acceptance (**b**) also only had a protective effect on males. Importantly, these gender effects need to be considered with caution due to unequal group size (230 females vs. only 77 males). Error bars indicate 95% confidence intervals of between-subject estimates. Boxplots denote 1st, 2nd, and 3rd quartiles with whiskers extending 1.5 inter-quartile ranges or until the most extreme data point has been reached. Data points beyond the whiskers are plotted individually.

For adaptive emotion regulation strategies (*CERQ-adapt*), we found a small but significant main effect of *time* (*F*(1, 291) = 5.61, *p* = 0.019, η_p_^2^ = 0.02), which was due to participants with less elaborated adaptive emotional regulation strategies experiencing stronger psychological *strain* (*r*s ≤  − 0.05). Beyond this main effect, we could reveal a three-way interaction of *CERQ-adapt*, *time*, and *gap* (*F*(1.77, 516.14) = 3.41, *p* = 0.039, η_p_^2^ = 0.01), which in turn was superseded by a four-way interaction with *gender* (*F*(1.77, 516.14) = 3.26, *p* = 0.045, η_p_^2^ = 0.01). Clarifying the four-way interaction, further analyses revealed that the three-way interaction of *CERQ-adapt*, *time*, and *gap* was only significant for male (*F*(1.79, 123.75) = 3.38, *p* = 0.042, η_p_^2^ = 0.05) but not for female participants (*F*(1.76, 391.25) = 0.54, *p* = 0.560, η_p_^2^ < 0.01). As can be seen in Fig. 3a, men with elevated adaptive emotion regulation strategies seemed to be able to buffer against psychological strain during pandemic onset only if the *gap* between assessments was high (*M* = 6.7 years, *SD* = 1.2 years: *r* =  − 0.36, *p* = 0.019) but not if it was low (*M* = 2.9 years, *SD* = 0.7 years: *r* = 0.17, *p* = 0.347). The baseline difference in strain between males with low compared to high adaptive emotion regulation strategies did not significantly vary as a function of *gap* (*r* =  − 0.18, *p* = 0.119).

Acceptance was treated as a separate predictor of the *CERQ* and did not show a significant main effect on psychological *strain* (*F*(1, 291) = 1.81, *p* = 0.180, η_p_^2^ < 0.01). However, a three-way interaction of acceptance, *time*, and *gender* emerged (*F*(1.77, 514.98) = 3.98, *p* = 0.024, η_p_^2^ = 0.01). As can be seen in Fig. 3b, only men seemed to benefit from acceptance, which buffered against the rise in *strain* that was observed in the whole sample during the first peak of the pandemic.

#### Childhood trauma

**Figure 4:**
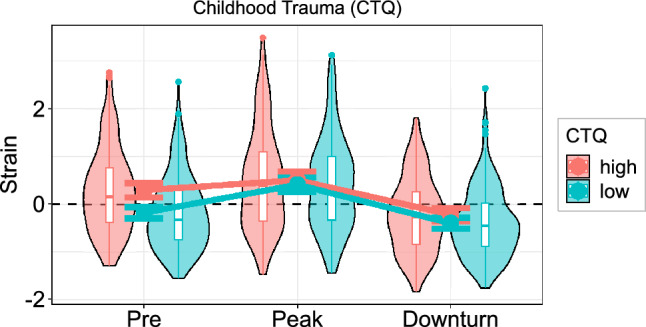
Effect of childhood trauma on psychological strain. Childhood trauma severity was associated with greater baseline strain and less increase until pandemic peak. Notably, compared to other risk factors (see Fig. 2), psychological strain during pandemic peak was independent of childhood trauma. Error bars indicate 95% confidence intervals of between-subject estimates. Boxplots denote 1st, 2nd, and 3rd quartiles with whiskers extending 1.5 inter-quartile ranges or until the most extreme data point has been reached. Data points beyond the whiskers are plotted individually.

Childhood trauma (*CTQ*) revealed similar effects as the risk factors described in Fig. 2. The main effect of the *CTQ* (*F*(1, 291) = 5.57, *p* = 0.019, η_p_^2^ = 0.02) denotes a generally positive association between childhood trauma severity and psychological *strain* but we also observed an interaction with time (*F*(1.79, 521.86) = 5.27, *p* = 0.007, η_p_^2^ = 0.02) that was driven by a baseline difference (*r* = 0.29, *p* < 0.001) followed by a reduced increase in individuals with higher *CTQ* (*r* =  − 0.25, *p* < 0.001), resulting in similar strain for all participants during peak pandemic that was independent of childhood trauma (*r* =  − 0.01, *p* = 0.913). The decrease in *strain* until pandemic downturn, however, was also smaller with increasing *CTQ* values (*r* = 0.12, *p* = 0.043) such that individuals showed small but significant differences in strain during the last assessment that could be predicted by childhood trauma severity (*r* = 0.12, *p* = 0.040; see Fig. 4).

#### Life events

Prior experience of threatening events (*LTE*) had no modulatory effects on the group-level results reported in Fig. 1 (*F*s ≤ 1.58, *p*s ≥ 0.210). Considering adverse life events (*ALE*), there were also no effects except for an unexpected and relatively weak five-way interaction of *ALE* × *time* × *gender* × *age* × *gap* (*F*(1.77, 515.81) = 3.22, *p* = 0.047, η_p_^2^ = 0.01). A description of this effect can be found in the Supplementary Materials.

## Discussion

In this (quasi-)longitudinal investigation of psychological burden across the COVID-19 pandemic in Germany, we found a medium negative effect on psychological wellbeing from before to the first peak of the pandemic (*d* =  − 0.40). Interestingly, this effect was counteracted by a large recovery during the relative downturn of the pandemic in fall 2021 (*d* = 0.93), which resulted in an overall *positive* effect of medium size compared to the pre-pandemic baseline (*d* = 0.44). This general pattern was moderated by social anxiety, childhood trauma, self-efficacy, and emotion regulation strategies: Participants with higher risk or lower protective factors experienced greater strain before the pandemic but also a *smaller* increase during its peak. Compared to men, female participants showed generally increased psychological burden independent of the pandemic and seemed to not benefit as much from adaptive emotion regulation strategies or acceptance. There were no clear patterns for threatening or adverse life events. Taken together, we obtained two unexpected results: There was an overall *positive* effect on psychological strain across the pandemic and a smaller initial increase for participants with *higher* pre-pandemic burden.

The first effect is in line with current research that found improvements in happiness^16^ and full recovery of life satisfaction^22^ across similar time frames throughout the pandemic. More specifically, our results were predominantly driven by participants with higher risk factors (social anxiety, low self-efficacy, maladaptive coping strategies; cf. Fig. 2) and could be explained by a shifting frame of reference in response to such an incisive event as a pandemic. These kinds of transformative challenges have already been described within survivors of (other) traumatic events. Calhoun and Tedeshi^41^ divide transformations of posttraumatic growth into three categories: changes in the perception of the self (strengths and new possibilities), experience of relationship with others, and one’s general philosophy of life (priorities, appreciation, and spirituality). Thus, in our case, individuals may have learned to appreciate the regained freedom again that they had taken for granted before lockdowns. Importantly, this change of reference due to incisive events seems to be independent of adaptive emotion regulation strategies (including reappraisal) since we did not observe clear effects for this moderator. Alternatively, the pandemic could have also stimulated social affiliation^42^. This perspective is consistent with improvements in perceived social support and interpersonal resources after having survived a mass shooting, which also predominantly occurred for individuals with elevated anxiety before the incident^43^. Crucially, it is currently unknown how persistent these outcomes will be. Future research should determine if such effects wear off quickly or change the perspective of individuals more sustainably.

Secondly, it appeared that risk factors of mental health impairments protected participants from an increase in psychological strain during the first peak of the pandemic to a certain extent. These results are in accordance with dampened responses in general distress and anhedonia-apprehension within individuals with higher neuroticism^37^. The interpretation of such results, however, is complicated by baseline differences in pre-pandemic burden, which are confounded with the prevalence of risk factors. Hence, it could be that the observed effect is simply a consequence of methodological particularities such as “regression to the mean”, the phenomenon that extreme values will likely be closer to the population average when measured again^44^. Keeping in mind that we acquired a nonclinical sample, however, it may also well be that relatively more strongly strained *healthy* individuals (in contrast to patients, cf.^11,13^) were better equipped to cope with the burden posed by the pandemic and thus experienced some kind of “home field advantage”. This interpretation is consistent with the mismatch hypothesis^45–47^, which states that individuals flourish best under circumstances that they are used to, even if these environments are adverse.

The main strength of the current study is the (quasi-)longitudinal examination of a relatively large and well-characterized cohort across the COVID-19 pandemic in Germany including a pre-pandemic baseline. However, some limitations also need to be acknowledged. First, we did not assess a single cohort throughout the pandemic but combined two samples to create a quasi-longitudinal trajectory (cf.^48^). Importantly, we only imputed the value during the first pandemic peak with the help of our second sample while the surprising effect of psychological strain dropping below the pre-pandemic baseline during pandemic downturn is comprised of true longitudinal observations. Hence, while the results with respect to the first pandemic peak may be affected by the quasi-longitudinal matching procedure, this is not the case for differences between before the pandemic and its downturn. Second, our sample exhibits a great variety with respect to the time when the first assessment was issued: The first participant was recruited in the middle of 2013 and the last one in the beginning of 2020. While the timing of assessment entails a trade-off between timeliness of pre-pandemic strain and contamination by first pandemic influences (e.g., news articles), we statistically controlled for potential effects of the time gap and only found interactions in combination with adaptive emotion regulation strategies as well as adverse life events. These effects, however, were very small in magnitude and just barely passed the alpha error threshold (*p*s ≥ 0.039, η_p_^2^ ≤ 0.01). On the other hand, this diversity in time gaps has the advantage that systematic influences of specific pre-pandemic events have been averaged out across participants, making our group-level estimate of pre-pandemic burden even more robust. Third, a problem for generalizability is posed by potential self-selection of participants. It can be expected that individuals with greater trust in the government and its regulations also showed more willingness to participate in a study conducted by a university. This subgroup may also have experienced less burden by the pandemic and associated governmental regulations. Such bias may be reflected by the relatively high number of 91% fully vaccinated individuals in our sample (compared to approximately 69% in the general population at that time^49,50^). Also, students were overrepresented at a fraction of 42%. Importantly, they may have retained more flexibility in following their occupation from home than employed individuals, which in turn may have positively influenced psychological wellbeing. Similarly, our sample was relatively young (*M* = 28.2 years) and due to the strict inclusion criteria free from mental disorders at the pre-pandemic time point. It might therefore be speculated that the current sample was more resilient than a representative community sample but it should be noted that we still observed large variability in psychological strain even in the current rather healthy participants and it has also been shown that younger populations seem to exhibit greater risk for psychological distress during the COVID-19 pandemic^9,13,14^. Lastly, females were overrepresented at 75%, which is why gender effects (especially higher order interactions for adaptive emotion regulation strategies or acceptance, cf. Fig. 3, but also the main effect over time, cf. Fig. 1) should be interpreted with caution. Taken together, since we observed no evidence for selective attrition, this lack of representation does not seem specific for the current research topic.

In summary, we found no evidence of long-lasting negative effects of the pandemic on the average trajectory of healthy people’s psychological strain. Individuals reporting low levels in known risk factors for mental health impairments or high levels in protective factors only showed short-lasting negative effects of medium size during pandemic peak. Pre-stressed participants, however, experienced a smaller decline of their psychological health that was even followed by a positive overcompensation during pandemic downturn. This indicates that healthy participants, on average, lived through the pandemic without permanent damage. Future research should evaluate the persistence of such compensatory relief effects in more detail.

## Materials and methods

### Participants

**Figure 5:**
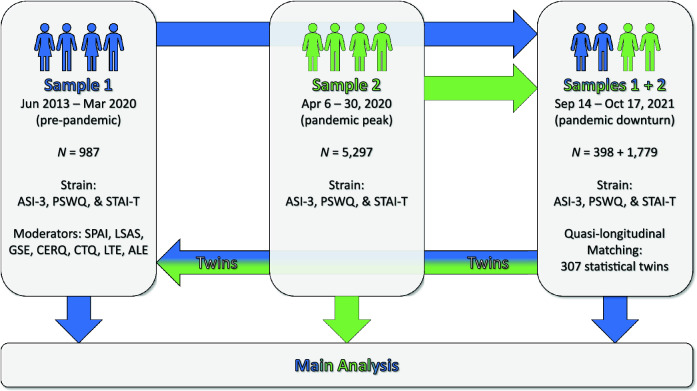
Overview of the acquired samples and analytical strategy. Sample 1 was assessed during the pre-pandemic baseline between June 2013 and March 2020 and a relative downturn of the COVID-19 pandemic in September and October 2021. Sample 2 was measured during the first peak of the pandemic in April 2020 and during the relative downturn in fall 2021. During all examinations, psychological strain was measured via a compound measure of the ASI-3, PSWQ, and STAI-T (cf. “Questionnaires” section). During the common measurement at the relative pandemic downturn, statistical twins were created (see Quasi-longitudinal Matching in “Data processing” section). The data of the twin from sample 2 was then used to impute the psychological strain during pandemic peak into the trajectory of the twin from sample 1, thus creating a quasi-longitudinal data set. For the main analysis, changes in psychological strain over time were analyzed relative to pre-pandemic moderators comprised of questionnaire sum scores for social anxiety (SPAI & LSAS), self-efficacy (GSE), emotion regulation strategies (CERQ), traumatic childhood experience (CTQ), and adverse life events (LTE & ALE).

Two independent samples were combined to allow for longitudinal inferences about the effect of the COVID-19 pandemic on mental health (see Fig. 5 for an overview). The first sample consisted of 987 individuals and was acquired prior to the COVID-19 outbreak between 2013 and the beginning of 2020 and had no current mental health diagnosis^51–53^. The second cohort was assessed during the first peak of the COVID-19 pandemic in Germany during April 2020 and included 5297 participants^54^. Since both samples granted permission to be contacted again for future studies, all individuals were invited to participate in a final survey during a relative downturn of the pandemic in fall 2021 (after the first wave of vaccinations had been rolled out^55^) in exchange for a 5% chance to win 50 €. Of the first sample, 398 individuals (40.3%) participated in the follow-up assessment, while 1779 individuals (33.6%) of the second sample accepted our invitation. After matching of participants (see details on the quasi-longitudinal matching below), 307 cases could be retained for analysis. The final sample consisted of 230 individuals who identified as female and 77 who identified as male. During the last assessment, mean age was 28.2 years (*SD* = 5.41 years, *range* = 18–50). All participants gave written informed consent. The study was approved by the local ethics committee of the Department of Psychology at the University of Würzburg and was performed in accordance with the Declaration of Helsinki.

### Questionnaires

#### Psychological strain

During every assessment, we asked participants to fill out the German versions of the Anxiety Sensitivity Index-3 (ASI-3^56,57^), the Penn State Worry Questionnaire (PSWQ^58,59^), and the trait version of the State-Trait Anxiety Inventory (STAI-T^60,61^). Cronbach’s α values were excellent (0.903, 0.927, and 0.937 respectively during the last assessment). To compute a composite outcome variable of psychological strain, we *z*-standardized all values of the ASI-3, PSWQ, and STAI-T (see Supplementary Materials for an exploratory factor analysis) to their mean and standard deviation of the pre-pandemic baseline and averaged the resulting *z*-scores into one index per participant and time point. This procedure has the advantage that the questionnaires provide equal contribution to the composite score while changes across the pandemic can be directly interpreted relative to pre-pandemic values. In summary, our measurement of psychological strain focusses on anxiety and depressive symptoms (cf.^62,63^).

#### Moderators

To predict how the trajectory of psychological strain was moderated by different protective and risk factors, we used the following questionnaires, which were only acquired during the pre-pandemic assessment: Social anxiety (cf.^19,23,24^) via the Social Phobia and Anxiety Inventory (SPAI^64,65^) and the Liebowitz Social Anxiety Scale (LSAS^66,67^); the Generalized Self-Efficacy scale (GSE^68,69^; cf.^29,30^); the short version of the Cognitive Emotion Regulation Questionnaire (CERQ-short^70,71^; cf.^31–33^) separated into maladaptive (CERQ-mal) and adaptive strategies (CERQ-adapt) as well as acceptance as a separate predictor (due to scientific disagreement about its classification; cf.^72,73^); and prior experience of adverse events (cf.^27,28^) via the Childhood Trauma Questionnaire (CTQ^74,75^), the List of Threatening Experiences (LTE^76^), and Adverse Life Events (ALE^39^) taken from the modified version of the Life History Calendar^77,78^. We initially aimed to explore further moderators from the last assessment like vaccination status, risk group membership, or previous COVID-19 infections but observed far too little variance for a systematic investigation: More than 90% of participants gave the same answer to these questions (cf. “Discussion” section on self-selection).

### Data processing

#### Longitudinal matching

For sample 1, 368 (92.5%) data sets could be retained. Twenty-nine (7.3%) subjects did not complete the questionnaire and for one participant, no pre-pandemic data had been acquired (i.e., a human error occurred when sending out invitations to the last assessment). For sample 2, 1604 (90%) data sets could be retained. The loss was caused by duplicates and inconsistencies in the provided anonymized code words. We checked unmatched codes for resemblance and manually rematched 290 data sets at face validity (see Supplementary Materials).

#### Quasi-longitudinal matching

Since the data before pandemic onset and during its first peak originated from independent samples (cf. Fig. 5), cases had to be united to provide an estimate for the full longitudinal trajectory of psychological strain across the COVID-19 pandemic. Therefore, we created statistical twins based on the survey of both samples during the pandemic downturn using multivariate matching (for an overview, see^79,80^). The data of the twin from sample 2 was then used to impute the data during pandemic peak into the data from its twin in sample 1, thus creating a quasi-longitudinal data set (cf.^48^).

To determine which variables are best suited for twin matching, we took an elastic net approach, which has been proven especially useful when relying on many predictors with an unknown covariance structure^81^. Critically, the elastic net balances model complexity and predictive performance by favoring variables that uniquely explain variance of the criterion. The result is a manageable set of distinctively meaningful predictors (cf.^54^). Data from sample 1 were subjected to the elastic net to predict the change from pre-pandemic strain to downturn by the multitude of variables acquired during the last assessment (see Supplementary Materials). According to the results, the change in strain was best predicted by depressive symptomatology (ADS-K and PHQ-2), inhibitory intolerance of uncertainty (IUS-I), and a single item describing the perceived change in one’s emotional mental state due to the COVID-19 pandemic within the last 6 months (i.e., spring to fall 2021).

We then submitted these four predictors alongside age and gender as key demographic variables and psychological strain as outcome measure to the “Match” function in R’s Matching package version 4.10-2^82,83^. We defined the maximum acceptable distance within twins to be 0.7 standard deviations for all variables. As a result, 42 female (15%), 17 male (18%), and two nonbinary participants (100%) from sample 1 could not be matched to a statistical twin from sample 2, yielding our final sample for analysis (*N* = 307; cf. Participants). Included participants showed high similarity to their statistical twins across matching variables (*r*s ≥ 0.93) with *z*-standardized differences averaging to 0.15 (*SD* = 0.18) for women and 0.18 (*SD* = 0.20) for men.

#### Main analysis

To analyze our data, mixed effects ANOVAs were computed with psychological *strain* as dependent variable, *time* point as within-subject factor, and the between-subjects predictors (a) *gender*, (b) *age* at last assessment, and (c) time *gap* between first and last time point. Further pre-pandemic predictors were added to the analysis one at a time. All continuous predictors were *z*-standardized before submitting them into the models. The Greenhouse–Geisser procedure^84^ was applied to correct for potential violations of the sphericity assumption in repeated-measures ANOVAs involving more than one degree of freedom in the numerator. Follow-up tests were performed two-sidedly at α = 5%, and corresponding effect sizes of Cohen’s *d* are reported with 95% confidence intervals around their point estimates. This procedure was not preregistered.

### Supplementary Information

Supplementary Information.

## Data Availability

The data that support the findings of this study are available upon reasonable request from the corresponding author: mario.reutter@uni-wuerzburg.de. The data are not publicly available because participants did not give written consent for their data to be shared publicly. Furthermore, the data contain sensitive, health-related information and enough information to potentially compromise the privacy of research participants.

## Code availability

All R code for data analysis is available on Github: https://github.com/spressi/Covid_burden.
