# Supplementary material for: Mental health improvement after the COVID-19 pandemic in individuals with psychological distress
Source: Sci Rep. 2024 Mar 7;14:5685. doi: 10.1038/s41598-024-55839-3 (PMC10920823; doi:10.1038/s41598-024-55839-3)
Supplement: Supplementary file 1 — Supplementary Information. [file 41598_2024_55839_MOESM1_ESM.pdf]

Supplement to:

# Mental health improvement after the COVID-19 pandemic in individuals with psychological distress

Mario Reutter[1], Katharina Hutterer[2], Marthe Gründahl[3], Dominik Gall[1], Udo Dannlowski[4], Katharina Domschke[5], Elisabeth J. Leehr[4], Tina Lonsdorf[6,7], Ulrike Lueken[8], Andreas Reif[9,10], Karoline Rosenkranz[6], Miriam A. Schiele[11], Dirk Schümann[6], Peter Zwanzger[12,13], Paul Pauli[1], Grit Hein[3*] & Matthias Gamer[1*]

[1] Department of Psychology, Julius-Maximilians-University Würzburg, Germany

[2] Center of Mental Health, Department of Psychiatry, Psychosomatics and Psychotherapy, Clinical Anxiety Research, University of Würzburg, Würzburg, Germany

[3] Center of Mental Health, Department of Psychiatry, Psychosomatics and Psychotherapy, Translational Social Neuroscience Unit, University of Würzburg, Würzburg, Germany

[4] Institute for Translational Psychiatry, University of Münster, Münster, Germany

[5] Department of Psychiatry and Psychotherapy, Medical Center – University of Freiburg, Faculty of Medicine, University of Freiburg, Freiburg Germany

[6] Institute for Systems Neuroscience, University Medical Center Hamburg-Eppendorf, Hamburg, Germany

[7] Department of Psychology, Biological Psychology and Cognitive Neuroscience, University of Bielefeld, Bielefeld, Germany

[8] Department of Psychology, Humboldt-Universität zu Berlin, Berlin, Germany

[9] Goethe University Frankfurt, University Hospital, Department of Psychiatry, Psychosomatic Medicine and Psychotherapy, Frankfurt, Germany

[10] Fraunhofer Institute for Translational Medicine and Pharmacology ITMP, Frankfurt, Germany

[11] Department of Psychiatry and Psychotherapy, Medical Center – University of Freiburg, Faculty of Medicine, University of Freiburg, Freiburg Germany

[12] kbo-Inn-Salzach-Klinikum, Clinical Center for Psychiatry, Psychotherapy, Psychosomatic Medicine, Geriatrics and Neurology, Wasserburg/Inn, Germany

[13] Department of Psychiatry, Ludwig-Maximilian-University Munich, Munich, Germany

* These authors contributed equally.

Corresponding author:

Dr. Mario Reutter

Department of Psychology I

Julius-Maximilians-University

Marcusstr. 9-11

97070 Würzburg, Germany

Phone: +49 931 31-84757

e-mail: mario.reutter@uni-wuerzburg.de

## Supplementary Materials and Methods

### Questionnaires

**Exploratory Factor Analysis.** We subjected the item-level data of the ASI-3, PSWQ, and STAI-T during the last assessment to an exploratory factor analysis using the nFactors package. Visual inspection of the scree plot (see below) and the acceleration criterion indicate a one-factor-solution. According to further indicators, extracting seven factors would also be possible but explained variance is already strongly diminished for factor two.

**Figure S1:** *Scree Plot with Decision Criteria*

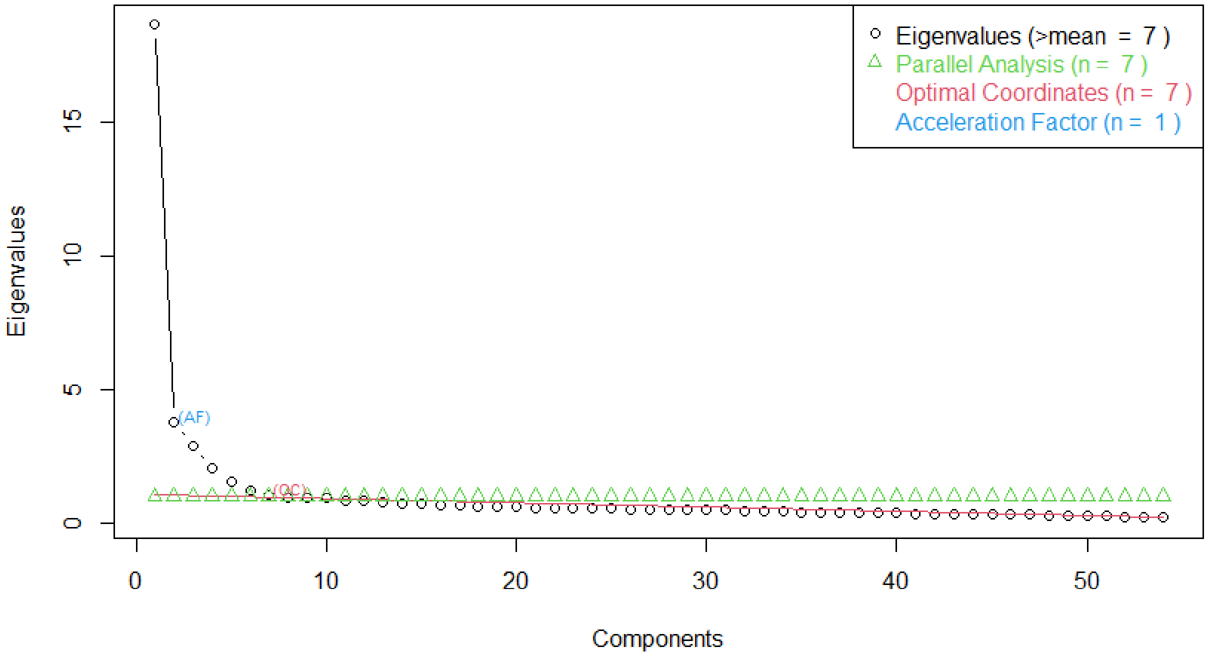


**Matching Variables.** To implement the quasi-longitudinal matching procedure described in the manuscript, the following variables were acquired during the last assessment at pandemic downturn and subjected to an elastic net regression model to predict the change from pre-pandemic strain to downturn:

1. The Connor-Davidson Resilience Scale (CD-RISC; Campbell-Sills & Stein, 2007; Wollny & Jacobs, 2023) with its total score and the subfacets flexibility, self-efficacy, self-regulation, optimism, and focus,
2. the Somatic Symptom Scale-8 (Gierk et al., 2014; Narrow et al., 2013),

3. the Fear of Covid-19 Scale (Ahorsu et al., 2022; Hein et al., 2021),
4. the two-item version of the Patient Health Questionnaire (PHQ-2; Löwe et al., 2005),
5. the state subscale of the State-Trait Anxiety Inventory (STAI-S; Laux et al., 1981; Spielberger et al., 1970),
6. the short version of the Intolerance of Uncertainty Scale (IUS-12; Carleton et al., 2007; Dietmaier et al., 2008) with its two factors Prospective Anxiety (IUS-P) and Inhibitory Anxiety (IUS-I),
7. the Illness Attitude Scales (IAS; Hiller & Rief, 2004; Kellner, 1987),
8. the Center for Epidemiologic Studies Depression Scale (CES-D Scale; Radloff, 1977) in its German short version "Allgemeine Depressionsskala Kurzform" (ADS-K; Hautzinger & Bailer, 1993),
9. and the Loneliness and Isolation during Social Distancing (LISD; Gründahl et al., 2022) with its two state factors ("lonely and isolated" & "connected and supported") and three trait factors ("loneliness and isolation", "sociability and sense of belonging", and "social closeness and support").

Furthermore, we acquired information on participants' vaccination status, belonging to a COVID-19-related risk group, prevalence of COVID-19 infections in the social environment, size of current household, and daily structure. We also asked subjects to estimate their perceived personal risk due to the COVID-19 pandemic as well as their perceived change in social and safety behavior, emotional functioning, sleep, and living circumstances due to the COVID-19 pandemic and associated restrictions within the last 6 months. Lastly, we asked participants to indicate their frequency of consuming media updates related to the COVID-19 pandemic and assessed demographic information like age, gender, citizenship, and occupation status.

As noted in the manuscript, the change in strain was best predicted by individual PHQ2, IUS-I, ADS-K, and a single item describing the perceived change in one's emotional mental state due to the COVID-19 pandemic within the last 6 months (i.e., spring to fall 2021).

## Data Processing

**Longitudinal Matching.** As mentioned in the manuscript, individuals of sample 2 participated anonymously, providing code words created from private information. During both assessments of sample 2, we asked participants to indicate their birthdate and the last two letters of the first names of each of their parents. If individuals gave inconsistent answers across time points, codes remained unmatched ($N = 465$). For every unmatched code of the last assessment, unmatched codes of the prior assessment were checked for concordance. Considering the two strings provided (one for each parent) and the three parts of the birthdate (day, month, and year separately), we calculated the number of mismatches across these five pieces. If a pair of codes had one or two mismatches and was the only pair with such high concordance, the pair was accepted preliminarily and flagged for manual inspection ($N = 262$ flagged; $N = 260$ accepted). For the remaining codes, all possible matches with the highest available concordance were manually screened. Further matches were accepted at face validity ($N = 203$). Examples of manual rematching include discrepancies that have occurred by a plausible typo (e.g., day "22" & "23"), typing the current year instead of one's birth year, or if participants seemed to have reported the *first* two letters of either parent during one occasion, enabling a plausible reconstruction of the parent's name (e.g., father "PE" & "ER" = Peter).

# Supplementary Results

Considering adverse life events (*ALE*), we found an unexpected five-way interaction of *ALE* × *time* × *gender* × *age* × *gap* ($F(1.77, 515.81) = 3.22$, $p = .047$, $\eta_p^2 = .01$). Individuals with an *age* below or equal to the median showed only a main effect of *time* ($F(1.78, 317.12) = 12.14$, $p < .001$, $\eta_p^2 = .06$) and a marginal main effect of life events ($F(1, 178) = 2.84$, $p = .094$, $\eta_p^2 = .02$). Participants with an age above the median exhibited a slightly more pronounced effect of *time* ($F(1.75, 198.15) = 14.94$, $p < .001$, $\eta_p^2 = .12$) but also an interaction of *time* and *gap* ($F(1, 113) = 6.23$, $p = .014$, $\eta_p^2 = .05$) and a marginal four-way interaction with

*time*, *gap*, *gender* and *ALE* ($F(1.75, 198.15) = 2.90$, $p = .064$, $\eta_p^2 = .03$). Follow-up analyses indicated that only older males showed the interaction between *ALE* and *gap* ($F(1, 40) = 5.30$, $p = .027$, $\eta_p^2 = .12$) while older females did not ($F(1, 73) = 1.65$, $p = .203$, $\eta_p^2 = .02$). Finally, this interaction could be traced back to the *ALE* only showing a tendency for a main effect for older males with a low *gap* between first and last assessment ($r = -.54$, $p = .086$) but not when *gap* between first and last assessment was high ($r = -.01$, $p = .964$).

## References


Ahorsu, D. K., Lin, C.-Y., Imani, V., Saffari, M., Griffiths, M. D., & Pakpour, A. H. (2022). The Fear of COVID-19 Scale: Development and Initial Validation. *International Journal of Mental Health and Addiction*, *20*(3), 1537–1545. https://doi.org/10.1007/s11469-020-00270-8

Campbell-Sills, L., & Stein, M. B. (2007). Psychometric analysis and refinement of the Connor-Davidson Resilience Scale (CD-RISC): Validation of a 10-item measure of resilience. *Journal of Traumatic Stress*, *20*(6), 1019–1028. https://doi.org/10.1002/jts.20271

Carleton, R. N., Norton, M. A. P. J., & Asmundson, G. J. G. (2007). Fearing the unknown: A short version of the Intolerance of Uncertainty Scale. *Journal of Anxiety Disorders*, *21*(1), 105–117. https://doi.org/10.1016/j.janxdis.2006.03.014

Dietmaier, G., Ille, R., Schäfer, A., Leutgeb, V., & Schienle, A. (2008). Kommentar zu Die Bedeutung von Unsicherheitsintoleranz für die Generalisierte Angststörung: Modellüberlegungen und Entwicklung einer deutschen Version der Unsicherheitsintoleranz-Skala. *Zeitschrift Für Klinische Psychologie Und Psychotherapie*, *37*(4), 272–275. https://doi.org/10.1026/1616-3443.37.4.272

Gierk, B., Kohlmann, S., Kroenke, K., Spangenberg, L., Zenger, M., Brähler, E., & Löwe, B. (2014). The somatic symptom scale-8 (SSS-8): A brief measure of somatic symptom burden. *JAMA Internal Medicine*, *174*(3), 399–407. https://doi.org/10.1001/jamainternmed.2013.12179

Gründahl, M., Weiß, M., Maier, L., Hewig, J., Deckert, J., & Hein, G. (2022). Construction and Validation of a Scale to Measure Loneliness and Isolation During Social Distancing and Its Effect on Mental Health. *Frontiers in Psychiatry*, *13*, 798596. https://doi.org/798596

Hautzinger, M., & Bailer, M. (1993). *Allgemeine Depressionsskala: (ADS)*. Beltz-Test-GmbH.

Hein, G., Gamer, M., Gall, D., Gründahl, M., Domschke, K., Andreatta, M., Wieser, M. J., & Pauli, P. (2021). Social cognitive factors outweigh negative emotionality in predicting COVID-19 related safety behaviors. *Preventive Medicine Reports*, *24*, 101559. https://doi.org/10.1016/j.pmedr.2021.101559

Hiller, W., & Rief, W. (2004). *Internationale Skalen für Hypochondrie: deutschsprachige Adaption des Whiteley-Index (WI) und Illness Attitude Scales (IAS)*. Verlag Hans Huber.

Kellner, R. (1987). *Abridged manual of the illness attitude scales*. University of New Mexico, Department of Psychiatry, School of Medicine.

Laux, L., Glanzmann, P., Schaffner, P., & Spielberger, C. D. (1981). *Das State-Trait-Angstinventar*. Beltz Testgesellschaft, Weinheim.

Löwe, B., Kroenke, K., & Gräfe, K. (2005). Detecting and monitoring depression with a two-item questionnaire (PHQ-2). *Journal of Psychosomatic Research*, *58*(2), 163–171. https://doi.org/10.1016/j.jpsychores.2004.09.006

Narrow, W. E., Clarke, D. E., Kuramoto, S. J., Kraemer, H. C., Kupfer, D. J., Greiner, L., & Regier, D. A. (2013). Dsm-5 field trials in the United States and Canada, Part III: Development and reliability testing of a cross-cutting symptom assessment for DSM-5. *The American Journal of Psychiatry*, *170*(1), 71–82. https://doi.org/10.1176/appi.ajp.2012.12071000

Radloff, L. S. (1977). The CES-D Scale. *Applied Psychological Measurement*, *1*(3), 385–401. https://doi.org/10.1177/014662167700100306

Spielberger, C. D., Gorsuch, R. L., & Lushene, R. E. (1970). The State-Trait Anxiety Inventory (Test Manual). *Consulting Psychologist*.

Wollny, A. I., & Jacobs, I. (2023). Validity and reliability of the German versions of the CD-RISC-10 and CD-RISC-2. *Current Psychology (New Brunswick, N.J.)*, *42*(5), 3437–3448. https://doi.org/10.1007/s12144-021-01670-2
